# Sexual dysfunction and related factors in pregnancy and postpartum: a systematic review and meta-analysis protocol

**DOI:** 10.1186/s13643-019-1079-4

**Published:** 2019-07-05

**Authors:** Mojdeh Banaei, Maryam Azizi, Azam Moridi, Sareh Dashti, Asiyeh Pormehr Yabandeh, Nasibeh Roozbeh

**Affiliations:** 1 Student Research Committee, School of Nursing and Midwifery, Shahid Beheshti University of Medical Sciences, Tehran, Iran; 2 Fertility and Infertility Research Center, Hormozgan University of Medical Sciences, Bandar Abbas, Iran; 3 Mother and Child Welfare Research Center, Hormozgan Universiy of Medical Sciences, Bandar Abbas, Iran; 4 Department of Community Health, Faculty of Medicine and Health Sciences, Universiti Putra Malaysia, 43400 UPM, Serdang, Selangor Malaysia

**Keywords:** Pregnancy, Sexual dysfunction, Women, Postpartum

## Abstract

**Background:**

Sexual dysfunction refers to a chain of psychiatric, individual, and couple’s experiences that manifests itself as a dysfunction in sexual desire, sexual arousal, orgasm, and pain during intercourse. The aim of this systematic review will be to assess the sexual dysfunction and determine the relevant factors to sexual dysfunction during pregnancy and postpartum.

**Methods and analysis:**

All observational studies, including descriptive, descriptive-analytic, case-control, and cohort studies published between 1990 and 2019, will be included in the study. Review articles, case studies, case reports, letter to editors, pilot studies, and editorial will be excluded from the study. The search will be conducted in the Cochrane Central Register, MEDLINE, Google Scholar, EMBASE, ProQuest, Scopus, WOS, and CINAHL databases. Eligible studies should assess at least one of the sexual dysfunction symptoms in pregnant women or in the first year postpartum. Quality assessment of studies will be performed by two authors independently based on the NOS checklist. This checklist is designed to assess the quality of observational studies**.** Data will be analyzed using Stata software ver. 11. Considering that the index investigated in the present study will be the level of sexual disorder, standard error will be calculated for each study using binomial distribution. The heterogeneity level will be investigated using Cochran’s Q statistic and *I*^2^ index in a chi-square test at a significance level of 1.1. Predictable limitations of this study included a small number and unacceptable quality of studies.

**Discussion:**

This systematic review addresses the factors associated with sexual dysfunction during pregnancy and postpartum. Considering the high prevalence of sexual dysfunction among women, the treatment of this problem has been highly sought after by the World Health Organization in recent years. The results of this study can help discover new strategies by introducing factors affecting women’s sexual dysfunction, thereby eliminating or diminishing these factors, and play an important role in improving the quality of life of women during pregnancy and postpartum periods.

**Systematic review registration:**

PROSPERO CRD42018083554

**Electronic supplementary material:**

The online version of this article (10.1186/s13643-019-1079-4) contains supplementary material, which is available to authorized users.

## Background

Sexual instinct is one of the strongest instincts of man that affects one’s behavior [1]. Sexual function, which is considered as part of the woman’s health, is an essential component of life and is a multi-dimensional phenomenon that is affected by many biological and psychological factors [2]. Sexual dysfunction refers to a chain of psychiatric, individual, and couple’s experiences that manifests itself as a dysfunction in sexual desire, sexual arousal, orgasm, and pain during intercourse [3]. Female sexual dysfunction (FSD) is one of the most common problems that affects about 40–45% of women [4].

Several risk factors affect the development of sexual dysfunction and sexual satisfaction of women, including mental health, sexual relations, female partner’s sexual function, and factors related to personality, duration of familiarity with the sexual partner, infertility, medications, chronic diseases, pelvic surgery, cancers, pregnancy, and postpartum period [5]. Sexual dysfunction leads to decreased quality of life and dissatisfaction with others, and negatively affects the physical, psychological, social, and emotional health of women [6, 7]. Disregarding this issue also leads to reduced sense of femininity, reduced self-confidence and security, and social problems including divorce, crime, drug addiction, and various mental and physical diseases [8].

Several factors, including hormonal changes, menstruation, pregnancy and childbirth, breastfeeding, menopause, and multiparity, affect sexual function [9]. Sexual feelings fluctuate throughout life. These fluctuations include transition due to pregnancy that results in changes in sexual function, which is considered as a psychosocial crisis. Generally, sexual function decreases during pregnancy and remains low in many women during the postpartum period [10]. Pregnancy and childbirth are a definite period in a woman’s life that causes hormonal and physical changes and has a significant effect on maternal health and quality of life [11].

Sexual and marital relationships change during pregnancy due to multiple physical and psychological changes. Factors including physiological and anatomical changes of a pregnant woman may affect sexual function in pregnancy. Some factors including abandoning sexual activity and feeling of guilt regarding sexual relations during pregnancy, altered body image, reduced sense of charm for the spouse, fear of injury to the fetus, fear of abortion, and early childbirth can affect woman sexual response and ultimately the couple’s relationship, leading to anxiety and lack of self-confidence in couples and eventually disrupting the mental health of the family [12]. Bayrami et al. showed that 66.3%, 50.7%, and 69.2% of women suffered from sexual dysfunction in the first, second, and third trimesters of pregnancy respectively, and sexual desire disorder will be the most commonly reported sexual dysfunction in each trimester of pregnancy [13]. Mother receives less attention and care during the postpartum period compared to the pregnancy period, and most deaths and disabilities occur during this period [14].

Postpartum sexual function is an important issue for couples, since the first postpartum sex is an important step for couples to establish sincere relationships [15]. Childbirth leads to anatomical and functional changes in the pelvic floor (PF) muscles, which might be responsible for some of women’s complaints about sexual problems during the postpartum period [6].

Several factors affect postpartum sexual dysfunction including the number of deliveries, breastfeeding, type of delivery, episiotomy, fatigue, and physical and psychological dysfunction including postpartum depression [16]. In the postpartum period, changes including pain during intercourse, lack of sexual desire, vaginal dryness, and failure to reach orgasm can affect the woman’s sexual response cycle. Sexual desire and sexual activity decrease during the postpartum period as compared to pregnancy, and sexual problems occur more frequently [17]. Studies have shown that 91.3% of women suffer from postpartum sexual problems [18].

The World Health Organization has recommended that a research must be conducted on sexual health, because of its importance, independently of reproductive health because lack of awareness about sexual health is the underlying cause of many dysfunctions and diseases worldwide [19]. The WHO has always emphasized that providing perinatal and postpartum care for mothers and babies and providing information and counseling to women in accordance to their needs is an ideal opportunity to address problems related to sexual health and sexual function [20].

Therefore, it is essential to identify various aspects of sexual problems during the postpartum period, know the indirect or indirect effects of this dysfunction on family relationships among couples, develop knowledge about sexual issues during the postpartum period, and highlight the importance of investigating their sexual issues during the postpartum period [21]. According to database search, no systematic review has been conducted on the factors associated with sexual dysfunction during pregnancy and postpartum so far. Systematic review studies summarize the reported results by explicitly stating the objectives and provide the best evidence for impartial judgment [22].

This systematic review will be conducted to assess sexual dysfunction and determine the relevant factors to sexual dysfunction during pregnancy and postpartum. Considering the high prevalence of sexual dysfunction among women, the treatment of this problem has been highly sought after by the World Health Organization in recent years. The results of this study can help discover new strategies by introducing factors affecting women’s sexual dysfunction thereby eliminating or diminishing these factors and play an important role in improving the quality of life of women during pregnancy and postpartum periods.

## Methods

### Study type

All observational studies, including descriptive, descriptive-analytic, case-control, and cohort studies published from 1990 to 2019, will be included in the study. Review articles, case studies, case reports, letter to editors, and editorials will be excluded from the study. There will be no language exclusion criteria nor any other publication restrictions.

### Type of participants

Observational studies will be selected to be included in this review study, if they met the following criteria:

1. Studies that included females older than 18 years old
2. Women should be pregnant or in the first year postpartum
3. Women should be diagnosed to have at least one of the sexual dysfunction symptoms
4. Lack of underlying diseases
5. Lack of pre-pregnancy untreated sexual problems
6. Lack of complications and problems with pregnancy
7. Lack of history of taking medications and supplements that affect sexual desire

### Type of exposure

Since different tools (exposures) will be used to measure sexual dysfunction in women, we include four exposures according to recent studies and finally we categorized the studies into subgroups. Types of exposures that will be included in this review will been based on the following criteria:

1. Use of Female Sexual Function Index (FSFI), Arizona Sexual Experiences Scale (ASEX), Golombok Rust Inventory of Sexual Satisfaction (GRISS), and Brief Index of Sexual Functioning for Women (BISF) to investigate the sexual dysfunction.

2. A total of 70% of participants should complete the study.

FSFI Questionnaire: FSFI is a 19-item questionnaire, which deals with sexual function in six domains of sexual function, including sexual desire (2 items), sexual arousal (4 items), lubrication (4 items), orgasm (3 items), satisfaction (3 items), and pain during sexual intercourse (3 items). These subcategories are scored based on a 5-point Likert scale, and a score higher than 5 refers to a better sexual function [23].

The ASEX questionnaire measures sexual function in five domains of sexual function, including sexual desire, arousal, lubrication/ penile erection, ability to achieve, and enjoy orgasm. The total score may range from 5 to 30, with a higher score indicating more sexual dysfunction [24]. The GRISS is a 28-item questionnaire for the assessment of sexual dysfunction which is consisted of 12 subscales, including impotence, premature ejaculation, anorgasmia, vaginismus, noncommunication, infrequency, male and female avoidance, male and female nonsensuality, and male and female dissatisfaction [25]. The BISF is a 22-item questionnaire adapted from the BSFQ for assessment of the frequency of sexual behavior, fantasy, masturbation, and sexual preference in women [26].

The Strengthening Reporting of Observational Studies in Epidemiology (STROBE) is a 22-item STROBE checklist, which will be used to investigate the standard reporting of studies. This checklist assesses, in the best possible way, the title and purpose of the articles, population and research samples, sampling methods, how the sources of bias were controlled for, the validity and reliability of the instruments used in the research, data analysis, results, and discussion of a study. The STROBE checklist divides studies into three levels, including weak, moderate, and strong [27, 28]. Studies that have obtained 70% of the checklist score [15] are included in the study.

### Primary outcome

The primary outcome will be to the determine the extent of sexual dysfunction in women. Sexual dysfunction will be measured using the FSFI, GRISS, BISF, and ASEX questionnaires.

### Secondary outcome

Secondary outcomes will be included:

1. Identifying sexual dysfunction in different domains
2. Determining the most common domain of sexual dysfunction
3. Determining factors associated with sexual dysfunction
4. Determining the most common domain of sexual dysfunction during the period of pregnancy and postpartum

### The search method used to identify studies

This strategy will include the search for published and unpublished studies. Databases which will be used include the Cochrane Central Register, PubMed, MEDLINE (Via PubMed), Google Scholar, EMBASE (Via Ovid), ProQuest, Scopus, WOS, and CINAHL. Keywords will be selected based on the MeSH terms and included "sexual problems", "sexual dysfunction", "sexual function," "pregnancy," "postpartum," "breastfeeding," "women," which will combine using Boolean “OR” and “AND” operators.

For each database, words and expressions will be chosen from controlled vocabulary (MeSH, EMTREE, and others) and free text searching. The search strategy will be designed by an information specialist. The details for the search strategy will be presented in Additional file 1. A snowballing method will be also used to identify other studies from the references of selected studies.

The initial search will be performed in MEDLINE and EMBASE using some of these keywords. Titles, abstracts, and keywords will be reviewed after text analysis. Then the search process will be performed in other resources using all the keywords. In addition to the databases, article references will be reviewed if they are relevant. Supplemental search will include a manual scan of the bibliographies of eligible studies, as well as gray literature including Conference Papers, Thesis and National reports, protocols and Key Journals including Sexual & Reproductive HealthCare, Sexual Health, Sexual Medicine, and Current Sexual Health Reports. Reporting checklist for search strategy will be based on PRISMA.

### Searching other resources

Manual search will be carried out in the following websites: https://www.unfpa.org/sexual-reproductive-health health and https://www.who.int/topics/sexual_health/en/ health.

### Data collection

#### Study selection

We use the Endnote X8.2 software to merge retrieved titles, remove duplicates, and screen titles and abstracts. Four investigators will review all potentially eligible citations to identify all relevant studies (two investigators (NR and AM) will perform the review, and two others (MB and MA) each review half of the identified studies). Two investigators (NR and MB) will perform a full-text review of selected citations to confirm study eligibility before extracting data. Disagreements will be resolved by consensus (NR, MB, AM, and MA).

### Data extraction

To extract data from the article texts, three authors independently will extract information using a researcher-made form. This form will be included the following items:

1. General characteristics (first author, date of publication, year of publication, referee’s name, article code, review date)
2. Study type (descriptive, descriptive-analytical, case-control, cohort, and longitudinal)
3. Sampling setting (hospital, community, health centers, clinics, etc.)
4. Sample size and target group
5. Sample characteristics (demographics, age, etc.)
6. Sample collection (sampling site and instruments used)
7. Result criteria (How the outcome variables were measured)

The fourth author will evaluate all data extraction forms independently, and in case of disagreement with other authors, an external referee will be approached.

### Quality assessment of studies

Two authors independently assessed the quality of the studies using the NOS checklist. This checklist is designed to assess the quality of observational studies. This instrument evaluates each study using eight items in three groups; selecting study groups, comparing groups, and proving the exposure or expected outcome. Stars are assigned to each of the approved quality items and the maximum score is 9 [29].

All studies will be scored based on this checklist, and the resulting score will present for each article in the form of a table. In case of discrepancies on the score assigned to published articles and to reach consensus, the discussion method and external referee will be used.

### Data analysis

Data analysis will be carried out using the STATA software version 11.

### Data entry and analysis

Quantitative data will be analyzed using Stata software version 11. Considering that the index investigated in the present study will be the level of sexual disorder, the standard error will be calculated for each study using binomial distribution. The heterogeneity level will be investigated using Cochran’s Q statistic and *I*^2^ index in the chi-square test at a significance level of 1.1. The random effects model will be used based on an inverse variance method to estimate the share ratio if the sample homogeneity hypothesis will be rejected. A forest plot will be used to display the results. Furthermore, moment-based metaregression will be used in order to investigate the effects of factors potentially affecting heterogeneity in the prevalence of sexual dysfunction. If possible, sub-group analysis will be performed based on the severity of sexual dysfunction for each domain, resumption of postpartum sex, and frequency of sex. The sexual dysfunction will be also analyzed based on parity and type of delivery. In circumstances where pooling of studies will be deemed inappropriate, we only provide a qualitative discussion of the findings. If data pooling will be appropriate based on low clinical diversity across studies (similar population and interventions), the outcomes will be pooled using Review Manager 5.3 (The Cochrane Collaboration, 2014). A risk ratio (RR) with 95% confidence intervals will be used to measure the effect in dichotomous and continuous variables respectively. We will use random effects models according to the DerSimonian-Laird method to take into account the underlying variation across studies [30].

## Discussion

This systematic review addressed the factors associated with sexual dysfunction during pregnancy and postpartum. Considering the high prevalence of sexual dysfunction among women, the treatment of this problem has been highly sought after by the World Health Organization in recent years. The results of this study can help discover new strategies by introducing factors affecting women’s sexual dysfunction thereby eliminating or diminishing these factors, and play an important role in improving the quality of life of women during pregnancy and postpartum periods.

## Additional file

Additional file 1: Search strategy. (DOCX 21 kb)

## Data Availability

Not applicable.

## Abbreviations

ASEX: Arizona Sexual Experiences Scale
FSD: Female sexual dysfunction
FSFI: Female Sexual Function Index
HUMS: Hormozgan University of Medical Sciences
PF: Pelvic floor
WHO: World Health Organization
