# Supplementary material for: Sexual dysfunction and related factors in pregnancy and postpartum: a systematic review and meta-analysis protocol
Source: Syst Rev. 2019 Jul 5;8:161. doi: 10.1186/s13643-019-1079-4 (PMC6612152; doi:10.1186/s13643-019-1079-4)
Supplement: Supplementary file 1 — Search strategy. (DOCX 21 kb) [file 13643_2019_1079_MOESM1_ESM.docx]

**Search strategy**

The Cochrane Library, ISI Web of Science, PubMed, EMBASE, MEDLINE, CINAHL, Scopus, Google Scholar, ProQuest, and Science Direct databases will be searched for relevant articles. Table summarizes the literature search strategy. After database search, the reference lists of the selected papers and relevant reviews will be evaluated and additional sources of information will be included accordingly. The review process is expected to begin on Des 1, 2018 and end within four months.

**Table 1.** PubMed and EMBASE and MEDLINE search strategy

|  | Search term |
| --- | --- |
| #1 | "Sexual Problem" [tiab] OR "Sexual Problems" [tiab] OR  "Sexual Dysfunction" [tiab] OR " Sexual Dysfunctions" [tiab] OR "Sexual Function" [tiab] OR " Sexual Behavior " [tiab] OR "Sexual Activity" [tiab] OR "Sexual Activities" [tiab] OR  " Sexual Disorder" [tiab] OR " Sexual Disorders " [tiab] |
| #2 | "Pregnancy"[tiab] OR "Pregnancies"[tiab] OR  "Gestation"[tiab] OR "Pregnant Women"[tiab] OR "Parturition"[tiab] OR " Parturitions"[tiab] OR "Births"[tiab] OR "Childbirth"[tiab] OR "Childbirths"[tiab] OR "Breast Feeding" [tiab] OR " Breastfeeding*"* [tiab] OR "Lactation"[tiab] OR "Postpartum Period"[tiab] OR "Postpartum"[tiab] OR "Puerperium"[tiab] OR "Postpartum Women"[tiab] |
| #3 | "Female"[tiab] OR "woman"[tiab] OR "women"[tiab] OR "Healthy women"[tiab] |
| #4 | "Prevalence"[tiab] OR "Epidemiology" |
| #5 | "Risk Factor"[tiab] OR " Risk Factors"[tiab] OR "Related Factor"[tiab] OR "Related Factors"[tiab] OR "Associated Factor"[tiab] OR "Associated Factors"[tiab] |

**Table 2.** Scopus search strategy

|  | Search term |
| --- | --- |
| #1 | TITLE-ABS-KEY ("Sexual Problem*" OR "Sexual Problems*"  OR  "Sexual Dysfunction*" OR "Sexual Dysfunctions*" OR "Sexual Function*" OR "Sexual Behavior*" OR "Sexual Activity*" OR "Sexual Activities*" OR  " Sexual Disorder*" OR " Sexual Disorders*") |
| #2 | TITLE-ABS-KEY ("Pregnancy*" OR "Pregnancies*" OR  "Gestation*" OR "Pregnant Women*" OR "Parturition*" OR " Parturitions*" OR "Births*" OR "Childbirth*" OR "Childbirths*" OR "Breast Feeding*" OR " Breastfeeding**"* OR "Lactation*" OR "Postpartum Period*" OR "Postpartum*" OR "Puerperium*" OR "Postpartum Women*") |
| #3 | TITLE-ABS-KEY ("Female*" OR "woman*" OR "women*" OR "Healthy women*") |
| #4 | TITLE-ABS-KEY ("Prevalence*" OR "Epidemiology*") |
| #5 | TITLE-ABS-KEY ("Risk Factor*" OR " Risk Factors*" OR "Related Factor*" OR "Related Factors*" OR "Associated Factor*" OR "Associated Factors*") |
